# The Exposure of Workers at a Busy Road Node to PM_2.5_: Occupational Risk Characterisation and Mitigation Measures

**DOI:** 10.3390/ijerph19084636

**Published:** 2022-04-12

**Authors:** Obuks A. Ejohwomu, Majeed Oladokun, Olalekan S. Oshodi, Oyegoke Teslim Bukoye, David John Edwards, Nwabueze Emekwuru, Olumide Adenuga, Adegboyega Sotunbo, Ola Uduku, Mobolanle Balogun, Rose Alani

**Affiliations:** 1 School of Mechanical, Aerospace and Civil Engineering, The University of Manchester, Manchester M13 9PL, UK; 2 School of Architecture, Building and Civil Engineering, Loughborough University, Loughborough LE11 3TU, UK; m.oladokun@lboro.ac.uk; 3 School of Engineering and Built Environment, Anglia Ruskin University, Chelmsford CM1 1SQ, UK; olalekan.oshodi@anglia.ac.uk; 4 Department of Information, Decisions and Operations, University of Bath, Bath BA2 7AY, UK; otb34@bath.ac.uk; 5 School of Engineering and the Built Environment, Birmingham City University, Birmingham B4 7XG, UK; drdavidedwards@aol.com; 6 Faculty of Engineering and the Built Environment, University of Johannesburg, Johannesburg 2092, South Africa; 7 Institute for Future Transport and Cities, Coventry University, Coventry CV1 5FB, UK; ab9992@coventry.ac.uk; 8 Department of Building, University of Lagos, Lagos 101017, Nigeria; oadenuga@unilag.edu.ng (O.A.); asotunbo@unilag.edu.ng (A.S.); 9 School of Architecture, University of Liverpool, Liverpool L69 7ZN, UK; o.uduku@liverpool.ac.uk; 10 College of Medicine, University of Lagos, Lagos 101017, Nigeria; mrbalogun@unilag.edu.ng; 11 Department of Chemistry, University of Lagos, Lagos 101017, Nigeria; ralani@unilag.edu.ng

**Keywords:** episodic event, elevated PM_2.5_ concentration, low and middle income countries (LMIC), occupational exposure, risk characterisation, control intervention, reference concentration

## Abstract

The link between air pollution and health burden in urban areas has been well researched. This has led to a plethora of effective policy-induced monitoring and interventions in the global south. However, the implication of pollutant species like PM_2.5_ in low middle income countries (LMIC) still remains a concern. By adopting a positivist philosophy and deductive reasoning, this research addresses the question, to what extent can we deliver effective interventions to improve air quality at a building structure located at a busy road node in a LMIC? This study assessed the temporal variability of pollutants around the university environment to provide a novel comparative evaluation of occupational shift patterns and the use of facemasks as risk control interventions. The findings indicate that the concentration of PM_2.5_, which can be as high as 300% compared to the WHO reference, was exacerbated by episodic events. With a notable decay period of approximately one-week, adequate protection and/or avoidance of hotspots are required for at-risk individuals within a busy road node. The use of masks with 80% efficiency provides sufficient mitigation against exposure risks to elevated PM_2.5_ concentrations without occupational shift, and 50% efficiency with at least ‘*2 h ON*, *2 h OFF*’ occupational shift scenario.

## 1. Introduction

Prior air pollution studies investigating occupational risk exposures of particulate matter (PM) have indicated a number of health concerns [1]. Increase in mortality rate (57%) is seen as the most common health-related consequence of air pollution to humans, while respiratory and cardiovascular diseases as a result of continuous exposure are also common (32.7% and 20.7%, respectively) [2,3,4]. In other words, outdoor PM is a major pointer to increase mortality rate in relation to cardiovascular issues [5,6]. Thus, exposure to PM through inhalation significantly changes the gut microbiota composition along the gastrointestinal (GI) tract. In cases where PM is inhaled, it gets deposited in the lungs through the following processes—impaction, interception, diffusion, and sedimentation [7,8]. Similarly, Wang et al. [9] found, in another study, that severe exposure to PM_2.5_ alters the composition of gut microbiota by causing gut dysbiosis and could ultimately result in the abnormal development in glucose metabolism. Whilst the yearly expenditure on diseases related to PM_2.5_ exposures is expected to reach about 5 billion yuan by 2030 due to cardiovascular and cerebrovascular admissions in hospitals in China [10], the implications of PM_2.5_ exposures in low and middle incomes countries (LMIC) still remains a concern [11]. For example, some parts of the city of Lagos evidently generate voluminous air pollution such as PM_2.5_ [12].

Commentators have argued that people, particularly within LMIC [13], do live and work in locations with high pollutant concentration [14]. For example, Lawin et al. [13] reported that an important part of the labour force in LMICs engage in commercial bus driving, cars, and motorcycles, where they are exposed to ambient air pollution. Obanya et al. [15] investigated air pollution around residential and transport sector locations (i.e., bus stops) in Lagos, Nigeria, and observed the respective concentration of PM_2.5_ and PM_10_ as 69.6 µg/m^3^ and 144.1 µg/m^3^, which is much higher than the WHO-recommended daily mean values of 15 µg/m^3^ and 25 µg/m^3^. These air quality measurements suggest that pedestrians are exposed to unacceptable levels of pollution when commuting through these locations and this has a direct causal link with health burden. 

Similarly, Ngoc et al. [16] posited that pedestrian exposure to particulate matter can be attributed to human activities, such as combustion of fuel, linked to cardiovascular and respiratory illness in people. While these scholars have examined a range of occupationally exposed risks within LMICs, prior work still offers very limited insights concerning the identification, analysis, and control interventions of occupational exposure risks within school environments. Amongst other factors, the accuracy of identifying air quality monitor depends largely on the instrumentation. Regardless, high-fidelity air quality monitoring stations are so expensive that their applications are limited [17]. This study employs an EarthSense Zephyr air quality low-cost sensor to measure the air pollution concentrations for effective analysis. Zephyr presents an ideal economical solution for the present study and can measure nitrogen oxide, nitrogen dioxide, ozone, particulates PM_1_, PM_2.5_ and PM_10_, temperature, and humidity [17]. 

Several control interventions have been developed and implemented to improve air quality in outdoor environments. Examples of interventions that are being implemented include: (1) discourage car idling [18]; (2) encourage the use of light rail transit [19,20]; (3) increase the uptake of electric and/or hybrid electric vehicles [21]; (4) congestion charging scheme [22]; and (5) replacement of vehicle exhaust system and use of face masks [23]. However, curiously, despite the importance of air quality and these interventions, little is known about the impact of these interventions in LMICs.

Thus, this study addresses this research question: to what extent can we deliver effective interventions to improve air quality at a building structure located at a busy road node in an LMIC? To address this RQ, our study sets out to achieve the following: (1) measure and characterise the pollutant concentration; (2) develop and assess the effective interventions to reduce exposure risk. The current study responds to the urgent need to identify effective strategies for reducing occupational exposure to particulates, such as PM_2.5_, in work environments [24,25]. The research draws on rich primary data collected from onsite measurement with a cloud-based air quality monitoring device.

This study offers three distinct contributions. First, the study measures and evaluates the occupational exposure to PM_2.5_ in the outdoor environments of a structure located at a busy traffic node. Thus, we presented both concentration levels and exposure risks based on WHO reference levels. Second, the findings provide a practical relevance which highlights the effectiveness of intervention strategies (such as occupational time shifts and use of personal protective devices) to reduce occupational exposure to PM_2.5_ in outdoor environments. Third, our study presents a novel methodological contribution by exploring time series measurements using a cloud-based instrument to determine occupational exposure to PM_2.5_ at a busy traffic node in a LMIC. 

## 2. Methodology

Set within this overarching epistemological context, a case study strategy was employed [26,27] and digital technologies were utilised to automate real time data acquisition [28,29]. A five-stage iterative research design process was then employed via: (1) establishing the experimental site; (2) research instrument set up; (3) uncertainty analysis of the measured variables; (4) temporal analysis of pollutant concentration; and (5) exposure risk characterisation and effects of control interventions.

### Measurement Site

**Figure 1:**
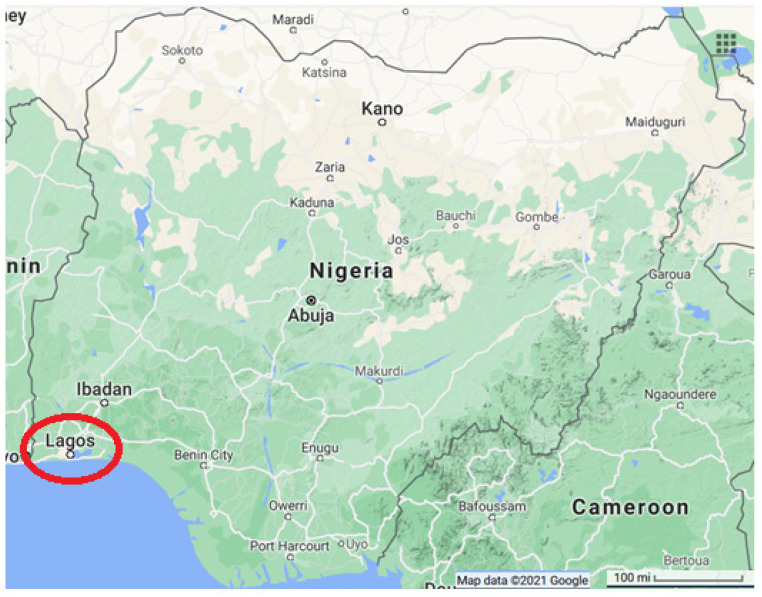
Lagos, highlighted, south of Nigeria in west Africa (Adapted from Google Maps (accessed on 24 August 2021)).

The series of measurements reported in this study took place at the main campus of the University of Lagos (6.5157° N, 3.3899° E), Lagos State, Nigeria. Lagos (see Figure 1) is the only city in Nigeria and West Africa approaching a mega-city status, with over 20 million residents [30]. With a large population, limited land mass, and associated high industrial, transportation, and other anthropogenic activities, the city generates voluminous air pollution such as PM_2.5_ [11]. 

**Figure 2:**
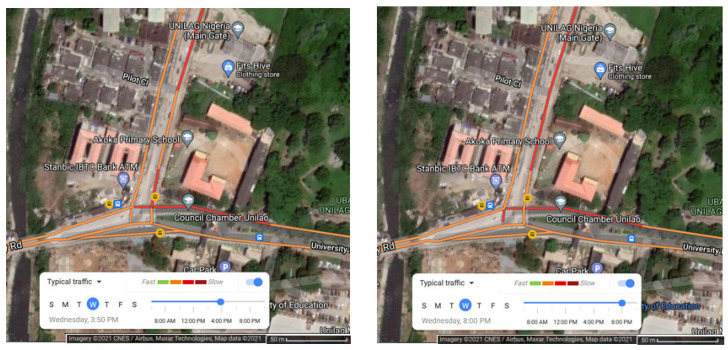
Typical traffic on a Wednesday at 4 p.m. (**left**) and 8 p.m. (**right**) on roads leading to the university gate house. Adapted from Google Maps (accessed on 24 August 2021).

The University of Lagos has a population of over 40,000 students and 4400 staff members [31,32]. The air quality monitoring device is located at the main gate house of the university which is situated at a busy traffic node between road intersections connecting outside vehicular and human traffic into the campus and vice versa. The main gate house consists of two gates, each enabling access into and out of the campus and holding at least six security operatives at any given time. There is a traffic roundabout 50 m in front of the gate house, where buses, commercial vehicles, and unauthorised vehicles to the campus can turn around without entering the gates. There is a bus stop within 50 m of the gate house. There are two twin-carriage roads leading into the campus entrance. Vehicular traffic volumes at a major ring road ~2 km from the main gate were, more than a decade ago, recorded at a weekday morning peak of 31,118 vehicles between 6 and 10 a.m. and an evening peak of 28,392 vehicles between 4 and 8 p.m. [33]. Figure 2 shows typical vehicular traffic in the afternoon and evening around the main gate. Additionally, about one in four of all undergraduate students (about 6250 students between 2007 and 2009) at the university either owned a vehicle or used one on campus [34]. At least six people are housed in the gate at any point in time as security operatives. 

**Figure 3:**
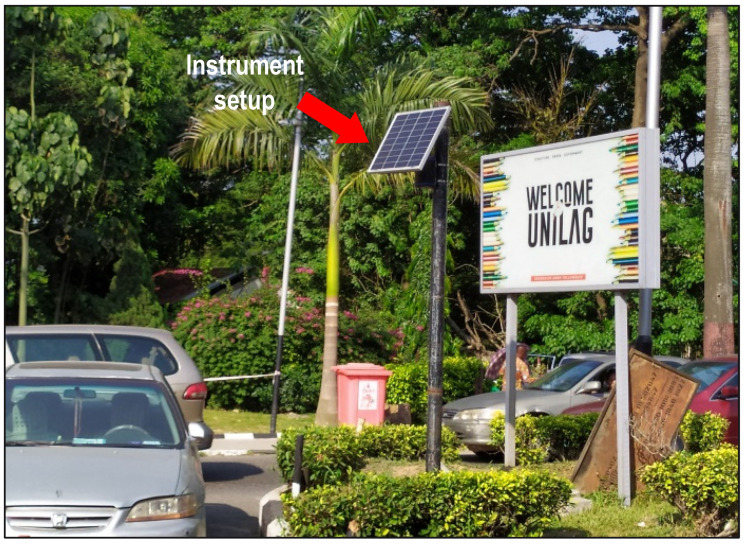
Measurement site at the university main gate house (© SQUARES Project).

Thus, the large academic and non-academic events within and around the university environment demand mobility of human and material resources and generates heavy transportation and pedestrian activities. Because heavy transportation and vehicular activities are associated with higher pollutant, especially PM_2.5_ concentration, the university gate provides an ideal location for assessing the pollutant concentration profile and the impact of control interventions on occupational exposure risk towards improving air quality at schools in low resourced countries. Figure 3 shows the measurement site as located near the road intersections at the main gate of the university main campus. 

## 3. Research Method

### 3.1. Instrumentation and Measurement Setup

Field measurements were randomly carried out at a busy road node from 22 December 2020 to 1 January 2021 using the EarthSense Zephyr air quality sensor. The sensor is pre-calibrated by the manufacturer by co-locating it with a local authority reference measurement to give accuracy of ±5 µg/m^3^ for PM_2.5_. The sensor, which combines on-board battery backup with solar power generation to avoid measurement interruption, was installed on a steel post (Figure 3) at about 2.5 m above the ground, with a clear wide space for the instrument to capture exposure of air pollutant affecting occupants around a building located at the busy road node. The setup of the instrument at this position is done to avoid measurement errors caused by illumination from the sun [35].

### 3.2. Uncertainty Analysis of the Measured PM_2.5_

The reliability of measured data depends on various uncertainties. For pollutant concentrations, these uncertainties range from those associated with the sensing equipment, installation of sensing equipment, logging system, correlation between the measured variable (e.g., temperature and PM_2.5_ concentration), and temporal fluctuation in the measurement [36]. These uncertainties can be reduced by the selection of an instrument with higher accuracy, good installation practices, and repeated measurement over an extended period [35]. The uncertainty in the PM_2.5_ concentration measurements were analysed per the guide to the expression of uncertainty in measurement [37]. In this approach, assuming a measured variable, X, consists of independent measurements $\;x_{1},\;x_{2},\cdots x_{n}$. The uncertainty in the variable can be estimated as a combined uncertainty $\;{∆}_{c}x$, with:

(1) $${∆}_{c}x=\sqrt{{\left(\frac{\sigma_{x,i}}{\sqrt{n}}\right)}^{2}+{\left(∆x,i\right)}^{2}}$$

where the first term on the right-hand side of Equation (1), $\sigma_{x,i}/\sqrt{n}$, is the standard uncertainty of the average measurement. $\sigma_{x,i}$ is the standard deviation of the measurement; n is the number of measurements; and ∆x,i is the accuracy of the measurement device as obtained from device manufacturer’s specification. For the PM_2.5_ concentration measured in this study, by substituting the accuracy of the PM_2.5_ sensor of ±5 µg/m^3^ into Equation (1), the uncertainty in PM_2.5_ measurement is 5.002 µg/m^3^. This suggests that the measurement is reliable within the instrumentation accuracy.

### 3.3. Temporal Analysis of Pollutant Concentration

The cloud-based sensor records time-series of pollutant concentrations at a frequency of one data point per minute, thereby resulting in 89,280 (1 × 60 × 24 × 62 = 89,280) data points over the two-month measurement period. However, the WHO [38] reference concentration levels are based on 24 h averaging windows. Thus, to facilitate the ease of comparison between the measured pollutant concentrations and the WHO reference concentration levels, the measured PM_2.5_ concentrations were pre-processed to 24-hourly averaging windows. 

### 3.4. Exposure Risk Characterisation and Effects of Control Interventions

To assess the occupational exposure risk to the PM_2.5_ concentration, the temporal risk characterisation ratio was computed for the pollutants. Risk characterisation ratio is a metric that compares the concentration at a measurement point to a standard reference concentration value [39]. It is computed as a quotient of the measured pollutant concentration to a standard reference value of the pollutant—refer to Equation (2). Similar metrics in air quality exposure risk assessment include intake fraction [40,41,42] that compare the concentration at occupant’s location with the source concentration and personal exposure index and/or susceptible exposure index [43,44] that compares pollutant concentration at the exhaust outlet of an enclosure to the one at the breathing zone of an exposed person. These metrics (i.e., intake fraction, personal exposure index, and susceptible exposure index) are similar because they compare the local concentration around an exposed person with a local concentration such as the emission source and concentration at exhaust outlet. In a situation, such as outdoor condition, where it is difficult to identify emission sources or exhaust outlets, risk characterisation ratio provides a better alternative. Risk characterisation ratio is computed from Equation (2) as:

(2) $$RCR_{pm2.5}=\frac{E_{mea}}{C_{ref}}$$

where $RCR_{pm2.5}$ is the risk characterisation of exposure to PM_2.5_; $E_{mea}$ is the exposure concentration due to the measured PM_2.5_ concentration (µg/m^3^); and $C_{ref}$ is the WHO reference concentration for PM_2.5_ (15 µg/m^3^). As a quotient of two concentration variables with the same unit of measurement (µg/m^3^), the risk characterisation ratio is dimensionless with its unit represented as (-). Expectedly, $RCR_{pm2.5}$ of values less than or equal to unity ($RCR_{pm2.5}\;\leq 1.0$) is of low/no risk level, while values above 1.0 are of higher occupational risk. Exposure is a mathematical product of pollutant concentration and the time over which a person is exposed to this concentration [45]. Exposure, therefore, involves an occurrence of two simultaneous events—a pollutant concentration at a particular place and time, and the presence of a person at that place and time. As such, to minimise exposure requires control of available concentration of pollutant, avoidance of locations of high pollutant concentration, or reducing the time of exposure to the concentration.

To control the available concentration of pollutant, such as PM_2.5_, source control forms the fundamental intent of many clean air policies. These include the public awareness against car idling, use of light rail transit, incentive on the uptake of electric and/or hybrid electric vehicles, congestion charging scheme, and replacement of vehicle exhaust system. However, where emission source control is insufficient, the use of personal protective devices, such as facemasks and other administrative and/or engineering controls, exist. This study considers the use of operational shifts and facemasks as respective administrative and engineering control interventions for minimising available concentration and reducing the time of exposure to the PM_2.5_ concentration. As pollutant concentration varies with time, exposure is typically calculated across the appropriate averaging time [44]. Thus, the exposure concentration, $E_{mea}$, in Equation (2) is computed as:

(3) $$E_{mea}=\left(1-P_{f}\right)\cdot \frac{1}{n}\overset{n}{\underset{i=1}{\sum }}C_{i}p_{i}$$

where $E_{mea}$ is the average temporal exposure concentration over the averaging window; $C_{i}$ is the temporal PM_2.5_ concentration; n is the averaging window (24 h for PM_2.5_); $P_{f}$ is the particle filtration efficiency of personal protective device, e.g., facemask; and $p_{i}$ is the presence of an exposed person at time, $t_{i}$., which is either one or zero to indicate that a person is present or absent at the time of concentration, $C_{i}$. As shown, Equation (3) accounts for occupational shift (with the presence factor, $p_{i}$) and the use of facemask with the $P_{f}$ parameter (ranging from zero for no mask to 99.9% for highly efficient facemasks). For instance, if a person is absent at a time, $t_{i}$, the exposure concentration becomes zero with $p_{i}$ value of zero. Similarly, for two people at a location with an average PM_2.5_ concentration of 35 µg/m^3^, where one of them uses no facemask (0% efficiency) and the other uses a facemask with 90% efficiency; the respective average exposure becomes 35 µg/m^3^ (i.e., $E_{mea}=\left(1-0\right)\times 35\times 1=35$) and 3.5 µg/m^3^ (i.e., $E_{mea}=\left(1-0.9\right)\times 35\times 1=3.5$).

To assess the impact of control interventions on exposure risk (defined by risk characterisation ratio, Equation (2)), this study considers the use of occupational shift and facemask as administrative and engineering measures, respectively. While the former involves ‘flexible shifts’ amongst the occupationally exposed persons at the test location, the latter involves the assessment of the effects of the use of facemask on exposure risk. Table 1 presents the intervention scenarios, where we consider five levels of facemasks with varying particle filtration efficiencies (i.e., $P_{f}$ in Equation (3)). It ranges from 5% (representing low efficient masks e.g., cloth mask) to 99% (representing highly efficient masks such as N95). Additionally, a case of zero percent mask Particle Filtration Efficiency (PFE) was considered to represent the control condition of no use of mask.

**Figure 4:**
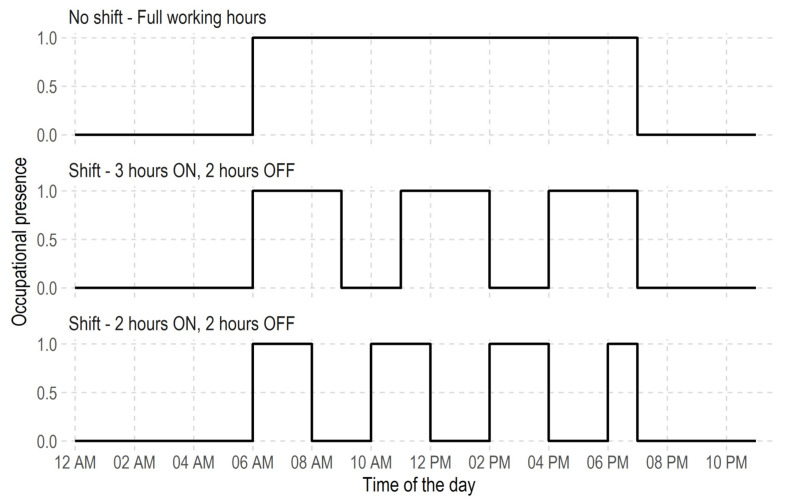
Hourly shift scenarios for exposure control intervention.

For the shift scenarios, two tests (Shift—2 h ON, 2 h OFF and Shift—3 h ON, 2 h OFF) and one control (No Shift—0 h) conditions were considered. In the shift scenarios, the ON/OFF conditions represent occupational presence where the $p_{i}$ value in Equation (3) is one and zero, respectively, for the ON and OFF conditions. While during the No Shift scenarios, an exposed person is present throughout the assessment period, for the Shift—2 h ON, 2 h OFF, the exposed person is present and absent at the location for 2 h respectively. During the Shift—3 h ON, 2 h OFF, however, the hypothetical person is assumed to be present for 3 h and absent for another 2 h. The present and absent values are defined as $p_{i}$ in Equation (3). Figure 4 shows the hourly shift scenarios for exposure control intervention considered in this study. Combining six (6) mask scenarios and three (3) shift scenarios, give 18 scenarios combinations (see Table 2). For each of the scenarios in Table 2, while the exposure concentration was computed using Equation (3), the risk characterisation is calculated with Equation (2).

Hypothetically, reducing exposure to or below the reference concentration level (such as defined by WHO [38]) provides effective intervention. Regardless, assessing the extent of delivering effective interventions requires the selection of a period of interest. For the test location, the mostly exposed populations are the security operatives working around the site. Hence, to assess the effect of the control interventions, two analytical procedures were defined. Firstly, we assumed the daytime working period of 6:00 a.m. and 6:59 p.m. for the security personnel, then assessed the exposure risk over this period of the day. Under these periods, the 0 h shift scenarios represent the presence of a staff over the whole working period of 6 a.m. to 6:59 p.m. Under the Shift—2 h ON, 2 h OFF shift scenarios, a security personnel is expected to have occupational presence for 2 h with a shift of 2 h in between each shift periods (see Figure 4). Similar conditions exist in the Shift—3 h ON, 2 h OFF shift scenarios, where a personnel is present for 3 h with a shift of 2 h in between each shift period (see Figure 4).

Secondly, we selected one week each from the months in the measurement periods. These periods were selected to cover the time of activities around the test location when the PM_2.5_ concentration may be high due to increased human and vehicular activities around the location. The selected periods include Christmas day, which represents social–religious activity, and examination days, representing academic events. With the peak concentration recorded on 27 December 2020 and 23 January 2021, the period for assessing the effects of control interventions is defined as 24–30 December 2020 and 20–26 January 2021. Thus, the dates (24–30 December 2020 and 20–26 January 2021) and time (6:00 a.m. to 6:59 p.m.) are used to subset the time-series data of PM_2.5_ exposure concentration for the assessment and analysis of control interventions. The main effects of each of the interventions defined in Table 2 were then examined in detail for their effectiveness in mitigating occupational exposure to pollutant concentration.

## 4. Results

### Profile of the Measured PM_2.5_ Concentration

Table 3 shows the summary statistics of the 15-min average PM_2.5_ concentration data collected over the two-month period. As shown, for the period of observation, the minimum PM_2.5_ concentration ranges between 10.53 µg/m^3^ and 12.27 µg/m^3^, while the maximum concentration ranges between 103.53 µg/m^3^ and 163.00 µg/m^3^. For both months of observation, the average concentration of PM_2.5_ of 25.43 to 29.38 µg/m^3^ exceeds the WHO reference value of 15 µg/m^3^, suggesting elevated concentration of PM_2.5_ at the test location.

**Figure 5:**
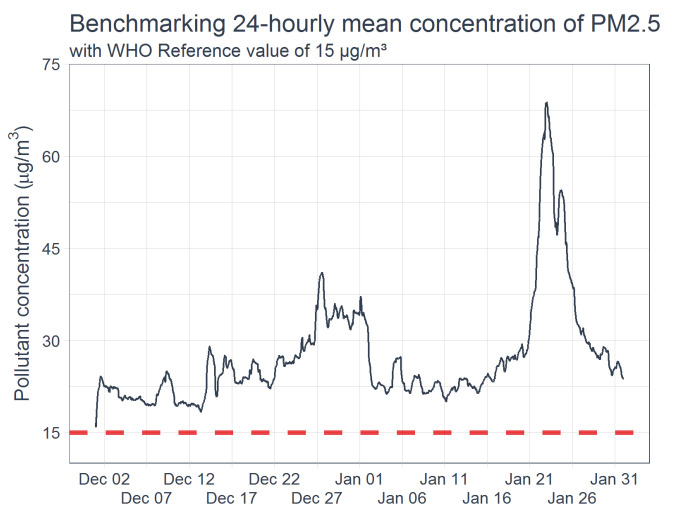
Measured PM_2.5_ concentration over the observation period compared with WHO referenced threshold.

Figure 5 compares the hourly temporal variation of the measured PM_2.5_ concentration with the WHO referenced value of 15 µg/m^3^. As shown, over the measurement periods, the PM_2.5_ concentration profiles exceed WHO reference concentration levels of 15 µg/m^3^ for the measurement periods. In those periods of high excitation, the PM_2.5_ concentrations can be as high as over four orders of magnitude.

**Figure 6:**
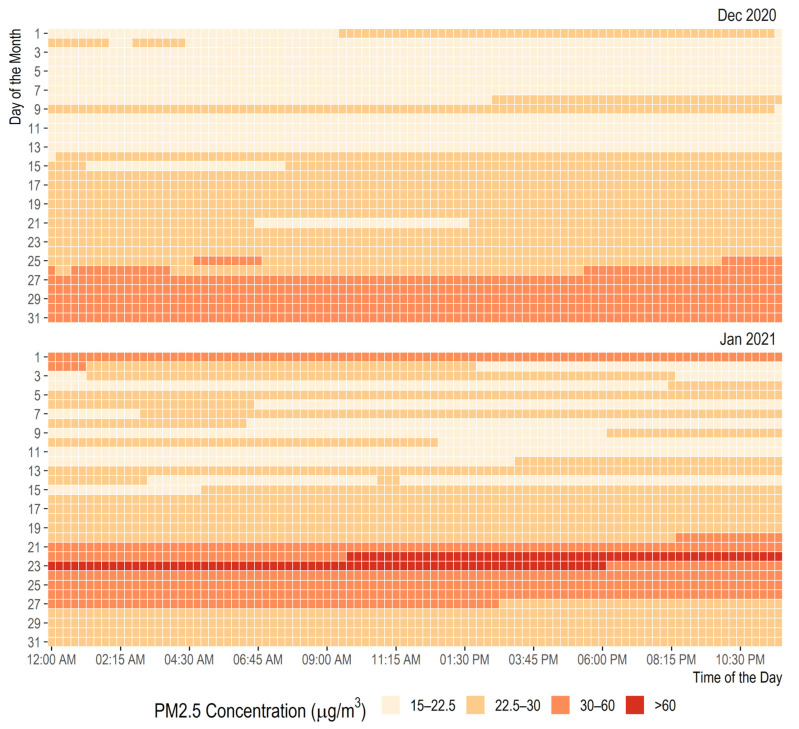
Hourly variation of PM_2.5_ concentration profile over the test period.

Higher levels of air pollution concentrations are related to more negative health outcomes [46]. As shown in Figure 5 above, PM_2.5_ have high episodic peak concentrations and such of high concerns for exposure assessments. Figure 6 shows the 24-hourly concentration of the PM_2.5_ profile over the test period. The results revealed that for most parts of the investigation periods, the concentration of PM_2.5_ ranges between 22.5 µg/m^3^ and 30.0 µg/m^3^ (about 1.5 to 2.0 order above the WHO reference values). On certain periods of the day, the concentration exceeds 60 µg/m^3^. This result raises a concern on the occupational exposure level of the exposed persons, especially the security personnel working around the test site. Hence, this study further assesses the influence of control interventions on the exposure level.

## 5. Results—Interventions

### Effects of Control Interventions on Exposure Risk

**Figure 7:**
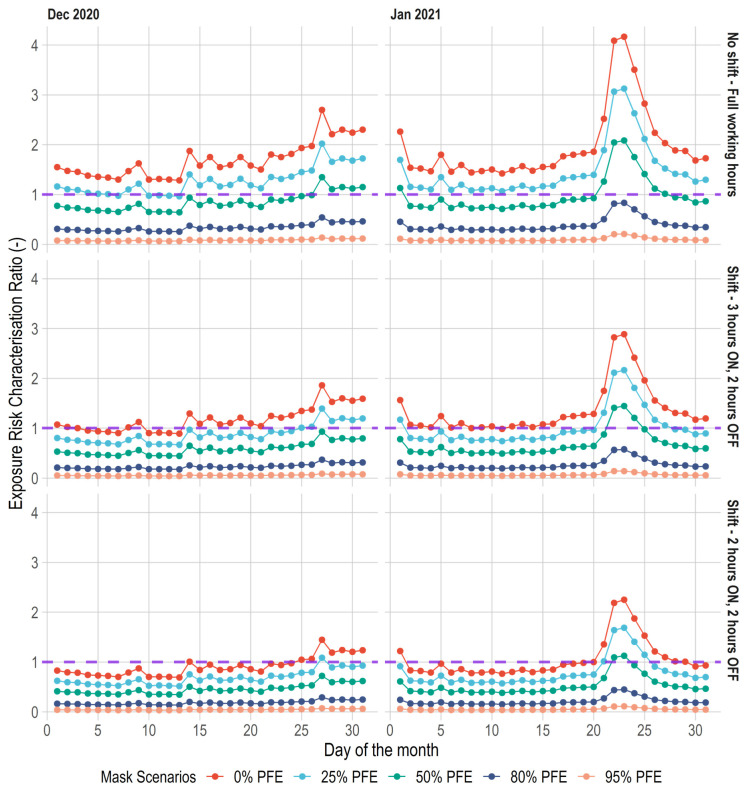
Influence of control interventions on daily occupational exposure risk.

Figure 7 shows the influence of control intervention on exposure risk profile to PM_2.5_ at the test location. The red dotted line on the graph indicates the *RCR* for PM_2.5_ based on the WHO reference exposure (i.e., based on the reference concentration) levels. As shown, the results indicate that the shift scenarios have significant influence on exposure risk. In the month of January, without the use of facemasks (i.e., 0% particle filtration efficiency), under the ‘*No Shift—Full Working Hours*’ the exposure risk is about 3 orders of magnitude above the WHO reference concentration. With ‘*Shift—3 h ON, 2 h OFF*’ and ‘*Shift—2 h ON, 2 h OFF*’ scenarios, the exposure risks were reduced to 2 and 1 orders of magnitude above the WHO reference value. Similar effects were obtained for the month of December.

These results suggest that occupational shifts reduce exposure risk to PM_2.5_ concentration. Additionally, shown on Figure 7 are the effects of the use of facemasks on exposure risks. As expected, the use of facemasks has a linear effect on exposure risks. However, irrespective of the shift scenarios, in certain days of elevated PM_2.5_ concentration, low efficient facemasks with 25% efficiency provide little protection against the risk of exposure to PM_2.5_ concentrations. Even with facemasks that have 50% efficiency, the protection against PM_2.5_ exposure risk is limited at elevated concentration such as observed on 23 January 2021. Conversely, regardless of the shift scenarios, higher efficient facemasks above 50% reduces exposure risks to PM_2.5_ concentration below the reference level of 1.0.

**Figure 8:**
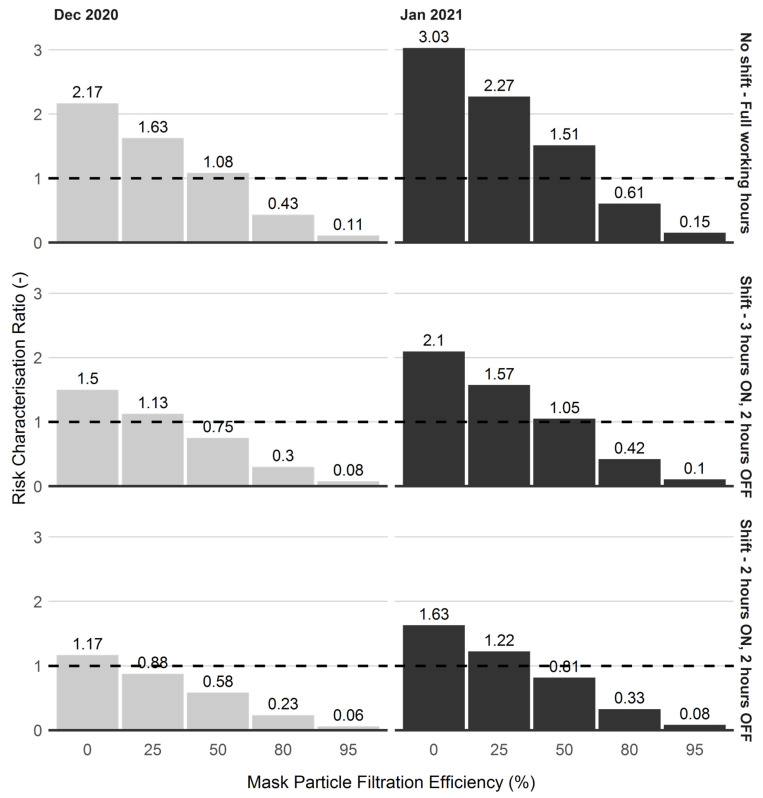
Effects of control interventions on occupational exposure risk over the period of socio-religious (24–30 December 2020) and academic (20–26 January 2021) events.

Figure 8 presents the distribution of effects of shift scenarios as well the use of facemask on exposure risk levels. Comparing between the levels of shift scenarios, the results show that the facemask of 25% efficiency is insufficient to reduce exposure risk level at or below the reference level. Additionally, when working at full hours without a shift, the facemask of 50% efficiency has about 51% risks over the reference level, suggesting its insufficiency to offer protection at elevated PM_2.5_ concentration. While the facemask of 50% efficiency offers marginal reduction of exposure to PM_2.5_ at ‘*Shift—3 h ON, 2 h OFF*’ scenario, its full benefits are revealed under the ‘*Shift—2 h ON, 2 h OFF*’ scenario. Under this scenario, a facemask of 50% efficiency reduces exposure by about 20% below the reference value. Findings from the control interventions suggest that short-time exposure with ‘*Shift—2 h ON, 2 h OFF*’ occupational shift offer reduction in exposure to PM_2.5_, with potential to improve this protection with the use of facemask with at least 50% efficiency. Although facemasks of efficiency higher than 50% (such as those of 80% and 95% efficiency) can further reduce the exposure risks, the benefit of using these facemasks becomes more beneficial when operational shift is infeasible. Where short-term exposure (such as ‘*Shift—2 h ON, 2 h OFF*’ scenario) is feasible, facemasks of 50% efficiency appear sufficient to reduce occupational exposure to PM_2.5_.

## 6. Discussion

Risks of exposure to PM_2.5_ concentrations at a busy road node have been assessed using on-site measurements. To meet the study objectives, a low-cost multi-pollutant air quality sensor was first used to measure and characterise the concentrations of PM_2.5_. Secondly, the study employed occupational shift and personal protection to assess the effectiveness of control interventions to reduce exposure risk to PM_2.5_ pollution. The influence of occupational shifts and use of masks to mitigate the risks of exposure to PM_2.5_ concentration were examined and analysed. This is followed by our primary findings.

With respect to the measured PM_2.5_ concentration considered in this study, the concentrations were significantly higher than the WHO reference concentration value. Over the two-month test periods, two episodic events of elevated concentrations were observed between 24–30 December 2020, and 20–26 January 2021. During these events, the average PM_2.5_ concentrations range between 25.4 and 29.4 µg/m^3^. A closer look into these periods revealed that the dates are related to social–religious and academic activities around the university. The elevated concentration in December is attributable to the Christmas celebrations, where a large section of the population goes for shopping, family visitation, and relaxation. The concentration began to increase at about two days before Christmas and continued until the beginning of January. Further, the episodic event in January, which occurred on the 23 January 2021, upon deeper analysis revealed that the period falls within the examination week in the university. As there were restrictions to on-campus accommodation due to COVID-19 pandemic, the movement of students and staff increased over this period, resulting in the elevated concentration (e.g., [31,32]). There is a common pattern in both observed episodic events—they span over many days. This will suggest that the decay period, i.e., the time to return to low level after the high concentration event (such as 27 December 2020 and 23 January 2021), can last for several days.

Regarding the effect of control interventions on exposure risks, occupational shifts seem to provide marginal protection at elevated concentration. This may be because exposure is estimated as a time weighted average and as the concentration is high over most of the periods changing the time of presence will provide little protection. As for the use of masks, considerable reduction in exposure risk is provided by masks above 50% efficiency. With the use of 80% and 95% efficiencies, the average exposure risks reduced to nearly zero values with ‘*Shift—2 h ON, 2 h OFF*’ occupational shift pattern.

Thus, this research has important implications to theory and practice. Our theoretical contributions are twofold and add to our understanding of occupational exposure risk characterisation and exposure of people to PM_2.5_ around buildings located at a busy road node. First, we extend prior studies on occupational exposure risk characterisation [1,2,5,6] by exploring the impact of occupational exposure risk characterisation of PM_2.5_ within LMICs. Second, extant studies on exposure of people to air pollutants, particularly PM_2.5_, have explored its impact on the abnormal development in glucose metabolism [9] and the cost implications of PM_2.5_ on hospital admissions [10]. Most recent studies have examined other health implications on residents across different age groups and indoor air quality [1]. We therefore extend the understanding and consequences of PM_2.5_ around buildings located at a busy road node.

This study offers relevant implications for organisations, policy makers, and stakeholders seeking to improve air quality around buildings located at a busy road node. We show the elevated concentrations over the decay period, which implies that there is a likelihood of higher exposure during social and academic events around the campus. Therefore, stakeholders, especially those with certain health concerns, should either avoid the environment or use personal protective equipment, such as facemasks, to reduce particle inhalation during this period.

Some limitations of this study are apparent and require further research. The measurements have been performed on a single station over a short period of time. These measurements sought to assess the temporal variability of pollutants around the university environment and to provide a first comparative evaluation of different control interventions. It was not the intention to accurately determine the long-term occupational exposure to pollutant such as PM_2.5_. The study acknowledges that exposed persons around the test locations can also be exposed at other locations, especially during non-working periods. However, capturing exposures other than the occupational setting (i.e., measurement site) is beyond the scope of the current study. Considering this additional information would require measurement at many locations for a period of several months. Importantly, the full-scale measurements of pollutant concentrations and assessment of the effectiveness of control interventions were only performed to assess the exposure risk levels around the university school gate, and performance of common control intervention to mitigate the risks.

Furthermore, the exposure risk characterisation is based on short-term 24-h mean reference concentration value of 15 µg/m^3^ for PM_2.5_. Future studies may focus on the long-term annual mean reference value of 5 µg/m^3^ for PM_2.5_, which is necessary for association of exposure to PM_2.5_ pollutions with health outcomes. The control interventions examined in this study have shown a fair reduction in exposure risks. This is because the measurements are recorded at a single location, whereas multiple measurements would provide more information to examine. However, the cumulative exposure with location shift in addition to time shift on single work location is considered in this study. Additionally, taking the exposure risk level due to the WHO reference concentration for PM_2.5_ as the target, the optimal mask scenario lies between the facemasks with efficiencies of 50% and 80%. Notably, the mask collection efficiency reported in this study is theoretical, as overall efficiency of face masks depends on many user-related factors in addition to the material-based variations presented in this study [3]. Regardless, comparing the filtration efficiency between scenarios is similar to mask efficiency under real-life application. Further, it is possible to optimise the interventions to determine the optimum mask efficiency at the most ideal shift scenario. The issue of optimisation is beyond the scope of the current study and could be explored in future studies. Vehicle-related interventions such as types and drive patterns (e.g., idling control) are good interventions to reduce vehicle-related emissions, but were not considered in the current study. Future studies may consider the impact of vehicle-related control intervention on the concentration and exposure risk mitigation around the campus gate, in particular, and the university environment in general.

## 7. Conclusions

A study of the influence of occupational shifts and use of masks on the risks of exposure to PM_2.5_ concentrations at a university school gate situated at a busy road node is presented in this paper. Time-series measurement of pollutant concentrations was conducted over a two-month period between December 2020 and January 2021 with a cloud-connected air quality sensor. The measurement uncertainty for the pollutants is within one percent of the instrument accuracy, thereby suggesting measurement reliability. The pollutant concentrations and exposure risks were characterised based on the WHO short-term 24-h mean reference concentration value. To assess the influence of the control interventions on mitigating exposure risks, the use of temporal shift scenarios and personal protective device with facemasks were employed. In the intervention analysis, the exposure risks were examined for both shift and mask scenarios over the test periods. The exposure risk characterisations for PM_2.5_ were evaluated by computing the quotient of temporal exposure concentration, with the WHO reference concentration value of 15 µg/m^3^. Thus, our findings are summarised as follows:

The concentration of particulate matters PM_2.5_ is found to be higher than WHO reference values. On certain periods relating to social–religious activities associated with Christmas celebration and academic activities around student examinations, the 24-h average concentration of PM_2.5_ can be as high as nearly 300% when compared with the WHO reference value of 15 µg/m^3^.

Following episodic events of elevated concentrations, the decay period can last for nearly one week, suggesting that adequate protections and/or avoidance of the environment is required for certain classes, especially the “at-risk individuals”.

The use of a personal protective device such as facemasks provided higher mitigation against exposure risks at elevated pollutant concentration than temporal shift scenario at the same location with high concentration.

With respect to mask scenarios, the use of masks with high efficiency, such as 80% and 95%, can provide little additional mitigation against exposure risks, especially at shorter occupational exposure of ‘*Shift—2 h ON, 2 h OFF*’ occupational shift. Considering the additional associated cost, the use of masks with 80% efficiency provides sufficient mitigation against exposure risks to elevated PM_2.5_ concentrations when there is no occupational shift, and 50% efficiency with at least ‘*Shift—2 h ON, 2 h OFF*’ occupational shift.

The outcomes of this study serve as a reference for future studies on the measurement and characterisation of urban air pollution and developing and/or assessing the effectiveness of control interventions in health risk assessments towards improving air quality and reducing occupational exposure risks. Future research may focus on, for example, long-term measurement at multiple locations within the university environment and coupling pollutant measurements with location-shift (in addition to time-shift at the same location), and vehicle-related interventions (vehicle types, drive patterns). Future research may also include measuring the long-term impact of exposure to air pollution on health outcomes of the populations around the university environment. Ultimately, LMICs need a paradigm shift in transportation policy towards battery technologies and green fuels. However, in the meantime, stakeholders’ engagement is required now to reduce the health burden that pollution has upon the local population, and this work serves to illustrate the magnitude of the issue, which calls for urgent action.

## Figures and Tables

**Table 1:** Scenario variables and their levels.

Scenarios	Levels
Mask Scenarios	PFE *—0%, 25%, 50%, 80%, 95%
Shift Scenarios	No Shift—0 h, Shift—2 h ON, 2 h OFF, Shift—3 h ON, 2 h OFF

* PFE: Particle Filtration Efficiency.

**Table 2:** Combination of scenario variables for assessing the effect of intervention on exposure.

Case-ID	Mask Scenarios	Shift Scenarios
1	pfe_00pct	shift_0 h
2	pfe_00pct	shift_2 h
3	pfe_00pct	shift_3 h
4	pfe_25pct	shift_0 h
5	pfe_25pct	shift_2 h
6	pfe_25pct	shift_3 h
7	pfe_50pct	shift_0 h
8	pfe_50pct	shift_2 h
9	pfe_50pct	shift_3 h
10	pfe_80pct	shift_0 h
11	pfe_80pct	shift_2 h
12	pfe_80pct	shift_3 h
13	pfe_95pct	shift_0 h
14	pfe_95pct	shift_2 h
15	pfe_95pct	shift_3 h

**Table 3:** Summary statistics of 15-min average PM_2.5_ concentrations data over two-month period.

Period	Statistics			
	Range (µg/m^3^)	Mean (µg/m^3^)	SD (µg/m^3^)	Median (µg/m^3^)
December 2020	[10.53, 103.36]	25.43	8.83	23.40
January 2021	[12.27, 163.00]	29.38	14.05	24.86

Note: Range = [Minimum, Maximum]; SD = Standard deviation.

## Data Availability

Not applicable.
